# Corrections to “An Optimized Safe Process from Bench to Pilot cGMP Production of API Eptifibatide Using a Multigram-Scale Microwave-Assisted Solid-Phase Peptide Synthesizer”

**DOI:** 10.1021/acs.oprd.2c00224

**Published:** 2022-07-29

**Authors:** Annunziata D’Ercole, Lorenzo Pacini, Giuseppina Sabatino, Matteo Zini, Lorenzo Milli, Francesca Nuti, Arianna Ribecai, Alfredo Paio, Paolo Rovero, Anna Maria Papini

Lorenzo Milli has been added to the author list.

